# BBS1 is involved in retrograde trafficking of ciliary GPCRs in the context of the BBSome complex

**DOI:** 10.1371/journal.pone.0195005

**Published:** 2018-03-28

**Authors:** Shohei Nozaki, Yohei Katoh, Takuya Kobayashi, Kazuhisa Nakayama

**Affiliations:** Graduate School of Pharmaceutical Sciences, Kyoto University, Sakyo-ku, Kyoto, Japan; University of Massachusetts Medical School, UNITED STATES

## Abstract

Protein trafficking within cilia is mediated by the intraflagellar transport (IFT) machinery composed of large protein complexes. The BBSome consists of eight BBS proteins encoded by causative genes of Bardet-Biedl syndrome (BBS), and has been implicated in the trafficking of ciliary membrane proteins, including G protein-coupled receptors (GPCRs), by connecting the IFT machinery to cargo GPCRs. Membrane recruitment of the BBSome to promote cargo trafficking has been proposed to be regulated by the Arf-like small GTPase ARL6/BBS3, through its interaction with the BBS1 subunit of the BBSome. We here investigated how the BBSome core subcomplex composed of BBS1, BBS2, BBS7, and BBS9 assembles and interacts with ARL6, and found that the ARL6–BBS1 interaction is reinforced by BBS9. *BBS1*-knockout (KO) cells showed defects in the ciliary entry of other BBSome subunits and ARL6, and in ciliary retrograde trafficking and the export of the GPCRs, Smoothened and GPR161. The trafficking defect of these GPCRs was rescued by the exogenous expression of wild-type BBS1, but not by its mutant lacking BBS9-binding ability. Our data thus indicate that the intact BBSome is required for retrograde trafficking of GPCRs out of cilia.

## Introduction

Cilia are specialized cell surface projections that function as cellular antennae by perceiving extracellular stimuli, such as fluid flow, and by receiving and transducing developmental signals, such as the Hedgehog (Hh) signal [1,2]. A cilium is composed of a microtubule-based scaffold called the axoneme, which is surrounded by a ciliary membrane that is continuous with the plasma membrane. Defects in ciliary assembly and functions therefore cause a variety of congenital disorders, such as Bardet-Biedl syndrome (BBS), Joubert syndrome, nephronophthisis, and Meckel syndrome, which are collectively referred to as ciliopathies [3,4]. These are pleiotropic disorders characterized by a broad spectrum of symptoms, including polycystic kidney, retinal degeneration, polydactyly, morbid obesity, and mental retardation.

The composition of proteins and lipids in cilia are greatly different from those of the cell body, because the transition zone at the base of cilia serve as a permeability/diffusion barrier [5,6]. Therefore, there are specific soluble and membrane proteins inside cilia and on the ciliary membrane. Intraflagellar transport (IFT) particles containing the IFT-A and IFT-B complexes are responsible for the trafficking of ciliary proteins [7–11]. The IFT-B complex mediates anterograde protein trafficking with the aid of kinesin-2 motors, whereas the IFT-A complex mediates retrograde trafficking powered by the dynein-2 complex [8–11]. In addition to the IFT-A and IFT-B complexes, the BBSome composed of eight BBS proteins moves along the axonemal microtubules in association with IFT particles [12] and has been implicated in the trafficking of ciliary G protein-coupled receptors (GPCRs).

We and others recently clarified the overall architecture of the IFT-B complex, which is composed of 16 subunits [13–15]. This very large complex can be divided into the core (B1) subcomplex composed of 10 subunits, and the peripheral (B2) subcomplex composed of 6 subunits; the two subcomplexes are connected by composite interactions involving two core subunits and two peripheral subunits. We also demonstrated the architecture of the IFT-A complex composed of 6 subunits, which associates with TULP3 [16].

We also clarified the architecture of the BBSome by taking advantage of the visible immunoprecipitation (VIP) assay [17], in which protein–protein interactions can be visually detected by analyzing whether an mCherry/TagRFP (mChe/tRFP)-fused protein is coimmunoprecipitated with an EGFP-fused protein. In the BBSome, four subunits, namely, BBS1, BBS2, BBS7, and BBS9 constitute the core subcomplex, whereas BBS4, BBS18, and BBS8 form the linker subcomplex; the two subcomplexes are connected by the interaction between BBS8 and BBS9 (see S1 Fig). BBS5 interacts with BBS9 and probably mediates the association of the BBSome with the ciliary membrane via its PH domain (see S1 Fig) [18]. In addition to these BBSome subunits, the Arf-like small GTPase ARL6/BBS3 was proposed to regulate the membrane recruitment and coat-like assembly of the BBSome via an interaction with BBS1 (see S1 Fig) [19–21]. Another BBSome-interacting protein, LZTFL1/BBS17, was also proposed to regulate BBSome function [22,23].

Nachury and colleagues proposed that the BBSome functions similarly to coat protein complexes, on the basis of the following reasons [19]: (i) All the core subunits of the BBSome have structural domains that are found in the subunits of coat protein complexes, including COPI, COPII, and clathrin-adaptor complexes. These domains include the β-propeller (BP) fold, γ-adaptin ear-homology (GAE)-like domain, and α/β-platform (PF) domain; (ii) similarly to the membrane recruitment of COPI, COPII, and clathrin-adaptor coats that is triggered by Arf/Sar1 GTPases, the BBSome is recruited onto synthetic liposomes through an interaction between BBS1 and GTP-bound ARL6; and (iii) the ciliary targeting sequence of SSTR3, which is a GPCR found on the ciliary membrane, is directly recognized by the BBSome.

In this study, to obtain clues toward understanding the association between the architecture of the BBSome and its function, we analyzed the interactions of the BBSome core subunits with one another, and furthermore, investigated the roles of BBS1 in the complex by establishing *BBS1*-knockout (KO) cell lines, followed by rescue experiments using wild-type (WT) and mutant BBS1.

## Materials and methods

### Plasmids, antibodies, and reagents

Expression vectors for BBSome subunits and their deletion mutants constructed in this study are listed in S1 Table. The antibodies used in this study are listed in S2 Table. Glutathione *S*-transferase (GST)-tagged anti-GFP nanobody (Nb) prebound to glutathione–Sepharose 4B beads were prepared as described previously [17]. Polyethylenimine Max and Smoothened Agonist (SAG) were purchased from Polysciences and Enzo Life Sciences, respectively.

### VIP assay and immunoblotting analysis

The VIP assay and subsequent immunoblotting analysis were carried out as described previously [14,17], with a slight modification; HEK293T cells expressing EGFP-tagged and mChe- or tRFP-tagged proteins were lysed in HMDEKN cell lysis buffer (10 mM HEPES [pH 7.4], 5 mM MgSO_4_, 1 mM DTT, 0.5 mM EDTA, 25 mM KCl, and 0.5% NP-40) [24]. Unless otherwise noted, the precipitated glutathione-Sepharose beads bearing fluorescent fusion proteins were observed using a BZ-8000 microscope (Keyence). The precipitated beads were also subjected to immunoblotting analysis, as described previously [17].

### Establishment of KO cell lines using the CRISPR/Cas9 system

The strategy for disruption of genes in hTERT-RPE1 cells (ATCC, CRL-4000) by the CRISPR/Cas9 system using homology-independent DNA repair (a version 2 method) was previously described in detail [25]; also see [16,24,26,27]. Briefly, single-guide RNA (sgRNA) sequences targeting the human *BBS1* gene (see S3 Table) were designed using CRISPR design [28]. Double-stranded oligonucleotides for these sequences were inserted separately into the all-in-one sgRNA expression vector peSpCAS9(1.1)-2×sgRNA (Addgene ID 80768). hTERT-RPE1 cells were grown on a 12-well plate to approximately 3.0 × 10^5^ cells, transfected with 1 μg of the sgRNA vector and 0.25 μg of the donor knock-in vector pDonor-tBFP-NLS-Neo(universal) (Addgene ID 80767) using X-tremeGENE9 DNA transfection reagent (Roche Applied Science), and cultured in the presence of G418 (600 μg/mL). Colonies of the cells carrying nuclear tBFP signals were isolated. Genomic DNA from these isolated cells was subjected to PCR using KOD FX Neo DNA polymerase (TOYOBO). Three sets of primers (S3 Table) were used to distinguish the three integration modes of the donor knock-in vector: forward integration (S2A and S2C Fig, b and b'), reverse integration (c and c'), and no integration with a small indel (a and a') (see [25]). Direct sequencing of the genomic PCR products was performed to confirm the disruption of the *BBS1* gene. Among the isolated *BBS1*-KO cell lines, we used the #B1-1-23 and #B1-2-21 lines in the present study; for detailed characterization of these cell lines, see S2 Fig.

### Preparation of cells stably expressing mChe-tagged BBS1 constructs

Lentiviral vectors were prepared as described previously [29]. Briefly, pRRLsinPPT-mChe-BBS1(WT) or its mutant was transfected into HEK293T cells using Polyethylenimine Max along with the packaging plasmids (pRSV-REV, pMD2.g, and pMDL/pRRE; kind gifts from Peter McPherson, McGill University [30]). Culture medium was replaced 8 h after transfection, and collected at 24, 36, and 48 h after transfection. The culture medium containing viral particles was passed through a 0.45-μm filter and centrifuged at 32,000 × *g* at 4°C for 4 h. Precipitated lentiviral particles were resuspended in Opti-MEM (Invitrogen) and stored at −80°C until use. *BBS1*-KO cells that express mChe-BBS1(WT) or its mutant were prepared by adding a lentiviral suspension to the culture medium.

### Immunofluorescence analysis

Induction of ciliogenesis and subsequent immunofluorescence analysis of hTERT-RPE1 cells were carried out as described previously [16,27]. The stained cells were observed using an Axiovert 200M microscope (Carl Zeiss). Statistical analyses were performed using JMP Pro 12 software (SAS Institute).

## Results

### Modes of interactions involving BBSome core subunits and ARL6

**Fig 1:**
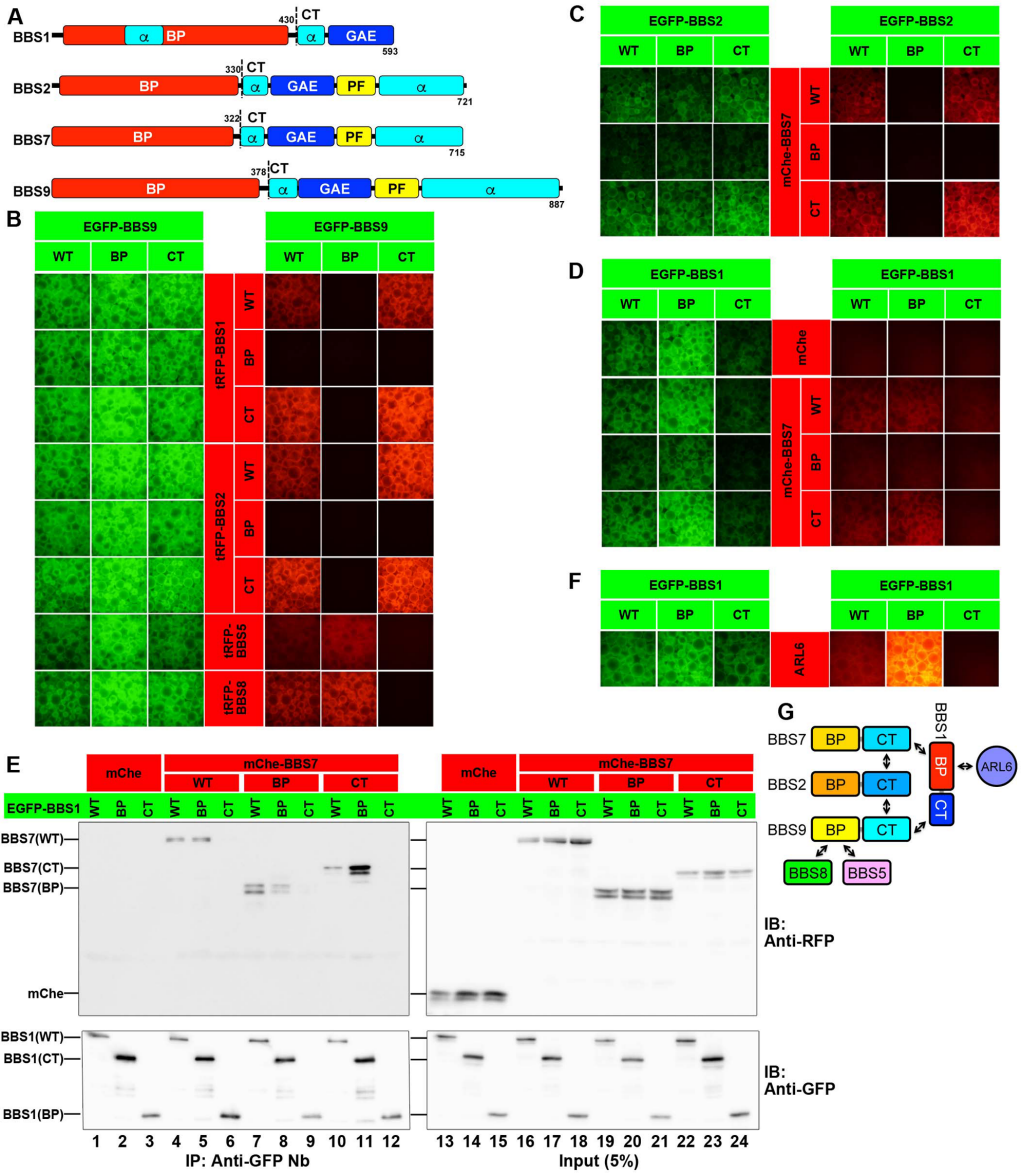
Modes of interaction involving BBSome core subunits and ARL6. (A) Schematic representation of the domain organizations of human BBS1, BBS2, BBS7, and BBS9. The amino acid positions of the boundary between the BP and CT constructs are also indicated. (B) HEK293T cells were cotransfected with expression vectors for a BBS9 construct fused to EGFP and a BBS1, BBS2, BBS5, or BBS8 construct fused to tRFP, as indicated. Lysates prepared from the transfected cells were subjected to the VIP assay, as described in Materials and Methods. (C) Lysates were prepared from HEK293T cells coexpressing an EGFP-fused BBS2 construct and mChe-fused BBS7 construct, as indicated, and subjected to the VIP assay. (D, E) HEK293T cells were cotransfected with expression vectors for an EGFP-fused BBS1 construct and mChe or an mChe-fused BBS7 construct, as indicated. Lysates were prepared from the transfected cells and processed for the VIP assay (D) or SDS-PAGE followed by immunoblotting analysis with an anti-RFP antibody, which reacts with mChe, or an anti-GFP antibody (E). (F) Lysates prepared from HEK293T cells coexpressing an EGFP-fused BBS1 construct, as indicated, and tRFP-fused ARL6∆N15(Q73L), were processed for the VIP assay. (G) Schematic representation of the predicted model of interactions involving BBSome subunits and ARL6.

In our previous study [17], we analyzed the 64 possible combinations of eight BBSome subunits tagged with EGFP and tRFP/mChe, by taking advantage of the VIP assay, and found that BBS1, BBS2, BBS7, and BBS9 constitute the core subcomplex (S1 Fig). These four core subunits share common domain organizations reminiscent of those of coat protein complexes [19]. All four subunits are predicted to have a BP fold in their N-terminal half (Fig 1A); for BBS1 and BBS9, their seven-bladed BP structures were confirmed by X-ray crystallography [31,32]. In their C-terminal half, these four subunits are predicted to have a GAE domain, which is followed by an α/β-platform (PF) domain (except for BBS1). Because BP folds are found in subunits of the COPI coat protein complex, which are subunits which participate in cargo recognition [33,34], and because GAE domains are found in clathrin adaptor proteins, the AP-1 γ-subunit, and GGA proteins [35], Nachury and colleagues proposed that the BBSome functions like a coat protein complex [19].

To delineate the interaction modes among the core subunits, we divided the WT BBS1, BBS2, BBS7, and BBS9 proteins into their N-terminal BP domain and their C-terminal (CT) region containing the GAE-like and α/β-PF domains, as schematically shown in Fig 1A, coexpressed them as EGFP- and mChe/tRFP-fusions in HEK293T cells, and subjected them to the VIP assay. In the VIP assay, protein–protein interactions can be visually detected by analyzing whether the mChe/tRFP-fused protein is coimmunoprecipitated with the EGFP-fused protein under a microscope [17]. As shown in Fig 1B, the BBS9 CT region fused to EGFP interacted with the tRFP-fused CT regions of BBS1 and BBS2 (row 3 and 6, respectively). On the other hand, the BP domain of BBS9 interacted with BBS5 and BBS8 (Fig 1B, row 7 and 8, respectively). The BBS2–BBS7 interaction was mediated by their CT regions (Fig 1C, bottom row).

The VIP assay suggested that the BP domain of BBS1 interacted mainly with the BBS7 CT region (Fig 1D, bottom row), although the BBS1 BP domain also exhibited an interaction with the BBS7 BP region (row 3). We also performed conventional immunoblotting analysis to confirm the VIP data, because a very recent study using the yeast two-hybrid system did not detect the BBS1–BBS7 interaction [36]. As shown in Fig 1E, the CT region of BBS7 made a major contribution to its interaction with the BBS1 BP domain (lane 11), although the BBS7 BP region also exhibited an interaction with the BBS1 BP domain (lane 8).

In agreement with a previous crystallographic study [32], ARL6∆N15(Q73L) was coprecipitated with the BP domain, but not the CT region, of BBS1 (Fig 1F); we here used an ARL6∆N15 (residues 1–15 deleted) construct, like in previous studies [19,32], as the presence of an N-terminal amphipathic helix generally hampers interactions of the ARF/ARL family GTPases with their effectors, at least *in vitro*.

Based on the data shown in Fig 1B–1F, we predicted the interaction model shown in Fig 1G. The BBS7–BBS2, BBS2–BBS9, and BBS9–BBS1 interactions are mediated by their CT regions, whereas the interaction between BBS1 and BBS7 is mediated mainly by their BP domain and CT region, respectively. The BBS1 BP domain also participates in its interaction with ARL6.

**Fig 2:**
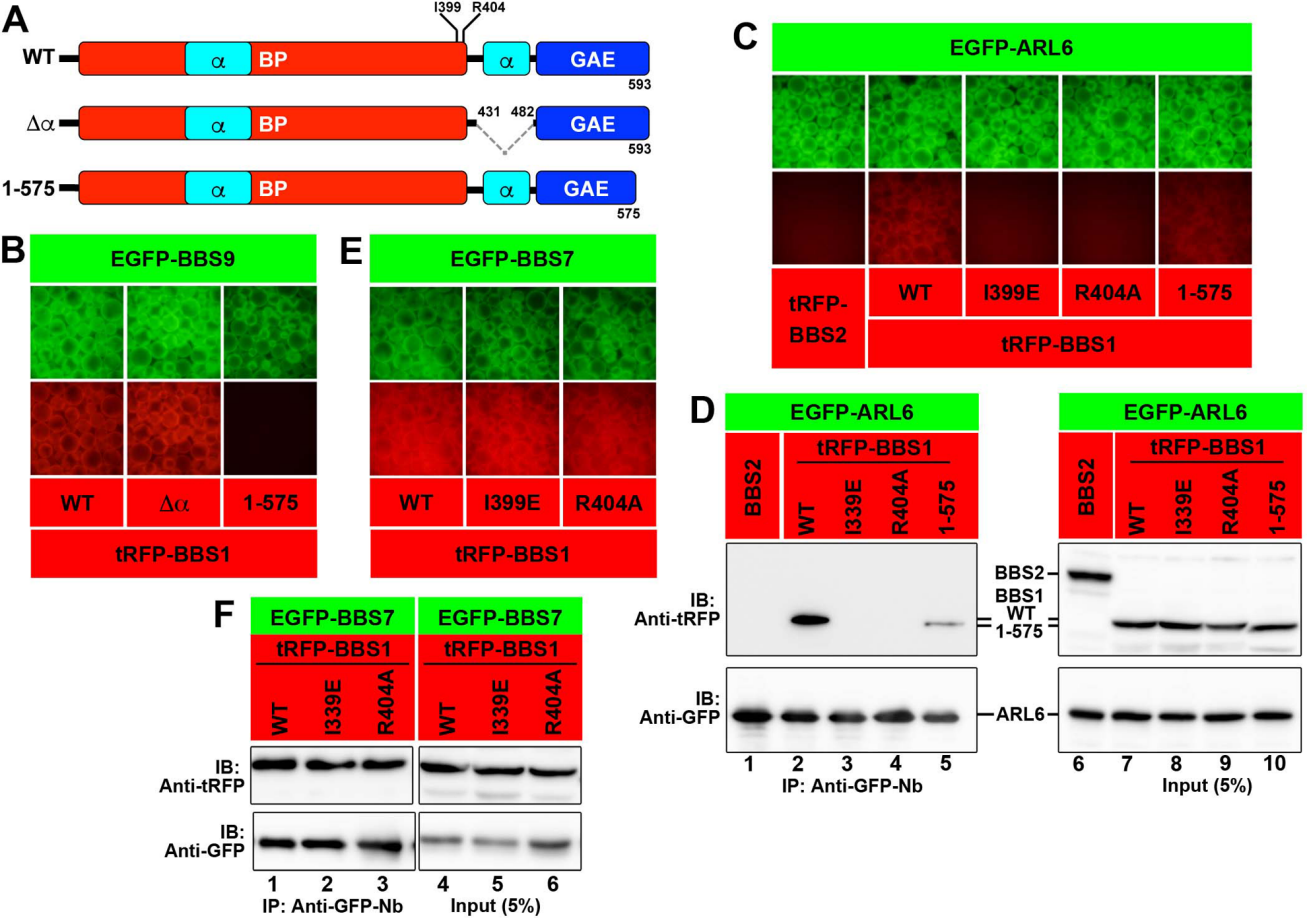
Interaction modes of BBS1 with BBS9, BBS7, and ARL6. (A) Schematic representation of BBS1 constructs used in the experiments. (B) Lysates prepared from HEK293T cells coexpressing the EGFP-BBS9 construct and mChe-fused BBS1 construct, as indicated, were subjected to the VIP assay. (C, D) HEK293T cells were cotransfected with expression vectors for EGFP-fused ARL6∆N15(Q73L) and a tRFP-fused BBS1 construct,as indicated. Lysates were prepared from the transfected cells and processed for the VIP assay (C) or SDS-PAGE followed by immunoblotting analysis with an anti-tRFP or anti-GFP antibody (D). (E, F) Lysates prepared from HEK293T cells cotransfected with expression vectors for EGFP-BBS7 and a tRFP-fused BBS1 construct as indicated were processed for the VIP assay (E) or SDS-PAGE followed by immunoblotting analysis with an anti-tRFP or anti-GFP antibody (F).

The CT region of BBS1 contains an α-helix region followed by a GAE-like domain. A BBS1 construct lacking the α-helix region (∆α; Fig 2A) retained the ability to interact with BBS9 (Fig 2B, column 2). By contrast, another BBS1 construct lacking the C-terminal 18-amino acids of the GAE-like domain (BBS1(1–575); Fig 2A) did not interact with BBS9 (Fig 2B, column 3). Thus, at least a part of the GAE-like domain of BBS1 participates in its interaction with BBS9.

Lorentzen and colleagues previously reported the crystal structure of *Chlamydomonas* ARL6∆N15 in complex with *Chlamydomonas* BBS1(1–425) [32]. They constructed some mutants of the human BBS1 BP domain on the basis of the crystal structure and found that an I399E or R404A mutation in human BBS1 abolished its interaction with ARL6; Ile^399^ and Arg^404^ are located within the bipartite blade 1 of the BBS1 BP domain [32]. By the VIP assay and conventional immunoblotting analysis, we confirmed their data; the I399E or R404A mutant of BBS1 could not interact with ARL6∆N15(Q73L) (Fig 2C and 2D, lanes 3 and 4). Somewhat unexpectedly, BBS1(1–575) demonstrated an attenuated interaction with ARL6∆N15(Q73L) compared with BBS1(WT) (Fig 2C and 2D, compare lane 5 with lane 2); this will be discussed later (see below). By contrast, both BBS1 point mutants retained their ability to interact with BBS7 (Fig 2E and 2F, lanes 2 and 3). Thus, it is likely that the BBS1 BP domain interacts with ARL6 and BBS7, at least in part via distinct interfaces.

### Impaired retrograde trafficking of ciliary GPCRs in BBS1-KO cells

Smoothened (SMO) and GPR161 are seven-pass transmembrane GPCRs involved in Hh signaling [1,2]. Under basal conditions, SMO is absent from cilia, whereas GPR161 on the ciliary membrane negatively regulates Hh signaling. When the Hh pathway is stimulated, for example, by treating ciliated cells with a small molecule activator, Smoothened Agonist (SAG), SMO enters cilia and GPR161 exit cilia; consequently, the negative regulation of the Hh signaling is canceled.

Sheffield and colleagues previously reported that, in cells derived from *Arl6*-KO mice, SMO is significantly accumulated within cilia even under basal conditions [20]. On the other hand, Nachury and colleagues reported that GPR161 was retained in the cilia of *Arl6*-KO IMCD3 cells even when the cells were treated with SAG [21]. These observations taken together suggested that retrograde trafficking and/or export of these ciliary GPCRs are impaired in the absence of ARL6.

**Fig 3:**
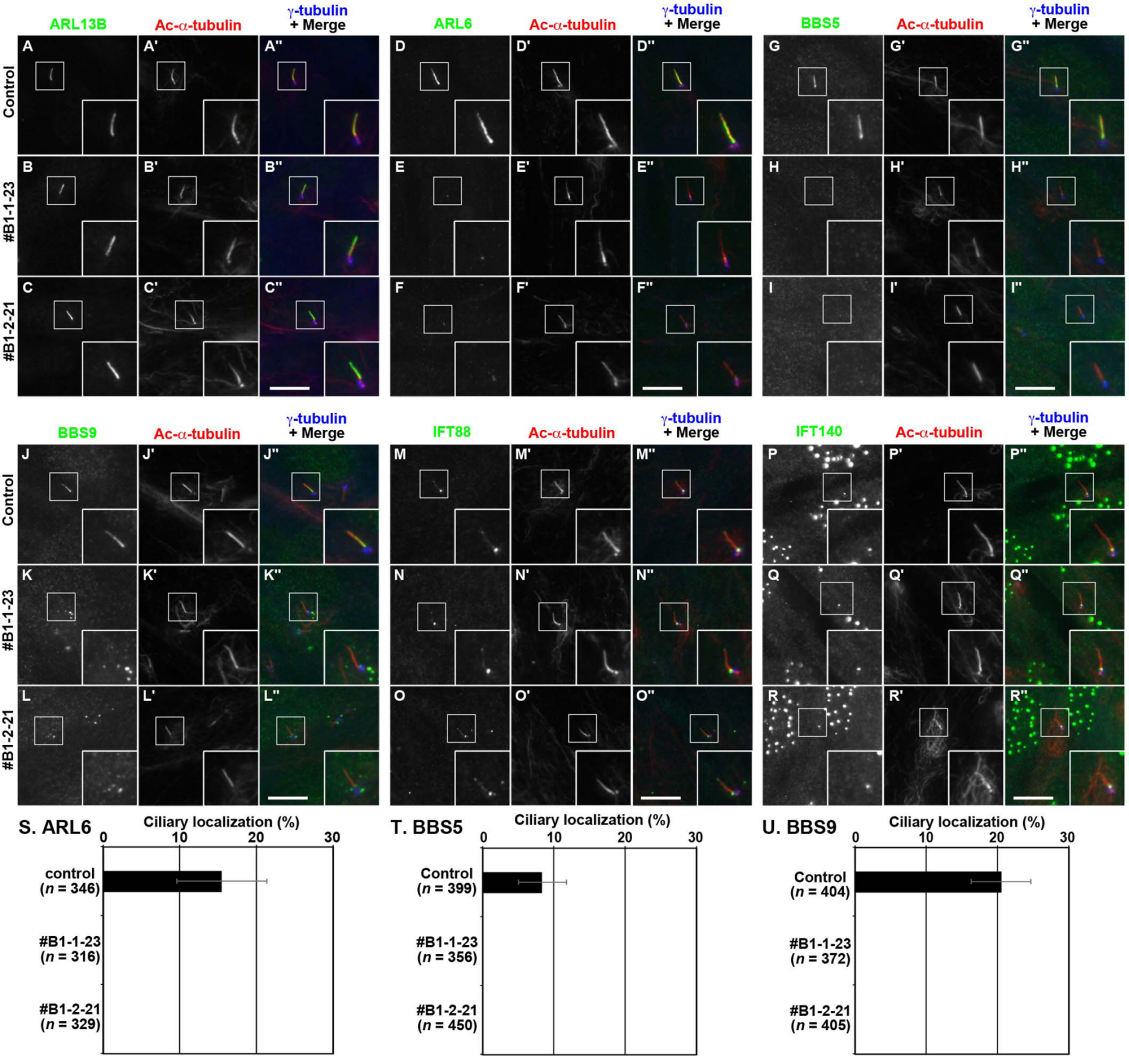
The BBSome and ARL6 do not localize within cilia in *BBS1*-KO cells. (A–R) Control RPE1 cells (A, D, G, J, M, and P) or the *BBS1*-KO cell lines #B1-1-23 (B, E, H, K, N, and Q) or #B1-2-21 (C, F, I, L, O, and R) were serum-starved for 24 h and triple immunostained for ARL13B (A–C), ARL6 (D–F), BBS5 (G–I), BBS9 (J–L), IFT88 (M–O), or IFT140 (P–R), and Ac-α-tubulin (A′–R′) and γ-tubulin (A′′–R′′). Insets show enlarged images of the boxed regions. Scale bars, 10 μm. (S)–(U), Control cells and *BBS1*-KO cells with ciliary localization of ARL6 (S), BBS5 (T), and BBS9 (U) were counted, and the percentages are expressed as stacked bar graphs. Values are means ± SD of three independent experiments. In each set of experiments, 82–152 (S), 109–185 (T), and 95–175 (U) ciliated cells were analyzed and the total numbers of ciliated cells analyzed (*n*) are shown.

In this study, we established *BBS1*-KO hTERT-RPE1 cell lines using a CRISPR/Cas9 system with our original modifications (the version 2 method; see [25]) and compared their phenotypes with those of control RPE1 cells, as ARL6 directly interacts with BBS1 and regulates BBSome function. Two *BBS1*-KO cell lines (#B1-1-23 and #B1-2-21) established using distinct target sequences were analyzed (see Materials and Methods and S2 Fig). Regarding localization of markers of the ciliary membrane (ARL13B) or the axoneme (acetylated α-tubulin; Ac-α-tubulin), no substantial differences were observed between control RPE1 cells and the *BBS1*-KO cell lines (Fig 3A–3C and 3A′–3C′). In addition, the frequency of ciliogenesis (S3A Fig) or ciliary length (S3B Fig) was not significantly different between control RPE1 cells and the two *BBS1*-KO cell lines.

ARL6 (Fig 3D), BBS5 (Fig 3G), and BBS9 (Fig 3J) were uniformly distributed within cilia in 10%–20% of control RPE1 cells (also see Fig 3S–3U). In marked contrast, localization of these BBS proteins was substantially altered in the *BBS1*-KO cell lines: ARL6 was no longer found inside cilia, but its localization at the ciliary base was, at least partially, retained (Fig 3E and 3F; also see Fig 3S); BBS5 localization within cilia and at the ciliary base was abolished (Fig 3H and 3I, also see Fig 3T); ciliary localization of BBS9 was also abolished, and BBS9-positive aggregates were often observed around the base (Fig 3K and 3L, also see Fig 3U). Although the identity of these aggregates is unclear, Sheffield and colleagues also reported the presence of BBS9-positive and BBS8-positive aggregates around the ciliary base in RPE1 cells treated with BBS1 siRNA [22]. Overall, it is thus likely that the BBSome and ARL6 cannot enter cilia in the absence of BBS1.

As the BBSome is believed to move within cilia in association with IFT particles containing the IFT-A and IFT-B complexes [12], we also analyzed the localization of IFT-A and IFT-B subunits in the *BBS1*-KO cell lines. However, the localization of IFT88 (an IFT-B subunit) or IFT140 (an IFT-A subunit) was not apparently altered in the absence of BBS1 compared with control RPE1 cells; IFT88 was mainly localized at the ciliary base with a minor proportion found along cilia (Fig 3M–3O), whereas the majority of IFT140 is found at the base (Fig 3P–3R). It is likely that intraciliary movement of the IFT-A or IFT-B complex is not dependent on the BBSome, although the BBSome moves in association with IFT particles.

**Fig 4:**
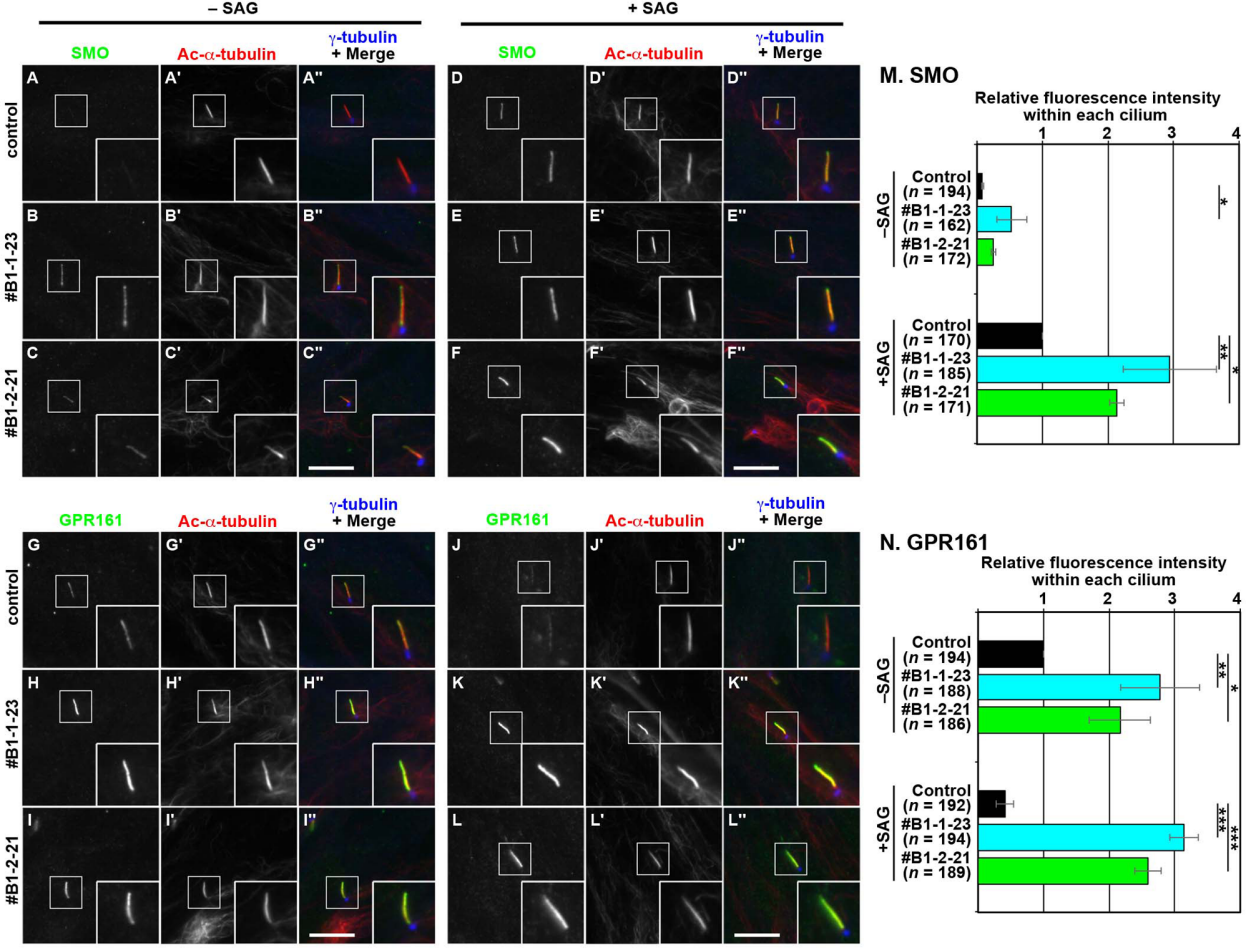
Accumulation of GPR161 within cilia in *BBS1*-KO cells. (A–L) Control RPE1 cells (A, D, G, and J), and the *BBS1*-KO cell lines #B1-1-23 (B, E, H, and K) and#B1-2-21 (C, F, I, and L) were serum-starved for 24 h and further cultured for 24 h in the absence (–SAG) or presence (+SAG) of 200 nM SAG. The cells were triple immunostained for either SMO (A–F) or GPR161 (G–L), Ac-α-tubulin (A′–L′), and γ-tubulin (A′′–L′′). Scale bars, 10 μm. (M and N) Fluorescence staining intensities of SMO (M) and GPR161 (N) in control and *BBS1*-KO cells were measured, and relative intensities of the cells, with SAG-treated control cells normalized to 1, are expressed as bar graphs. Values are means ± SD of three independent experiments. In each set of experiments, 50–71 (M) and 50–69 (U) ciliated cells were analyzed and the total numbers of ciliated cells analyzed (*n*) are shown. *, *p* < 0.05; **, *p* < 0.005; ***, *p* < 0.0001 (one-way ANOVA followed by Tukey post-hoc analysis).

We then compared the localization of SMO and GPR161 under basal (–SAG) and SAG-treated (+SAG) conditions. Under basal conditions, the ciliary localization of SMO was not detected in control RPE1 cells (Fig 4A), but was substantially increased in the *BBS1*-KO cell lines (Fig 4B and 4C, also see Fig 4M). Upon stimulation with SAG, SMO entered cilia in control RPE1 cells (Fig 4D, also see Fig 4M), and its ciliary localization was further enhanced in the *BBS1*-KO cells (Fig 4E and 4F, also see Fig 4M). These observations suggest two possibilities: one is that the BBSome suppresses ciliary entry of SMO; and the other is that, even under basal conditions, SMO undergoes constitutive cycling between the ciliary and plasma membranes to keep its ciliary level low, and that a block in its retrograde trafficking and/or exit from cilia in the absence of BBS1 might result in significant retention of SMO on the ciliary membrane. In view of the GPR161 data (see below), we favor the latter possibility.

On the other hand, in control cells, GPR161 was found within most cilia under basal conditions (Fig 4G), and its localization to cilia was significantly decreased under SAG-stimulated conditions (Fig 4J). Under basal conditions, localization of GPR161 within cilia in the *BBS1*-KO cell lines (Fig 4H and 4I; also see Fig 4N) was significantly higher than that in control cells (Fig 4G). Furthermore, unlike in control cells, GPR161 was retained within cilia even when the *BBS1*-KO cells were treated with SAG (Fig 4K and 4L; also see Fig 4N). The altered localization of SMO and GPR161 in the *BBS1*-KO cells under basal and SAG-stimulated conditions is reminiscent of our previous study on cells lacking IFT139, which is a subunit of the IFT-A complex [16]; in *IFT139*-KO cells, retrograde trafficking and/or export of ciliary GPCRs, including SMO and GPR161, was severely impaired. These observations together indicate that, in the absence of BBS1, retrograde trafficking and/or ciliary exit of the GPCRs involved in Hh signaling is impaired.

### BBS1 mediates retrograde trafficking of GPCRs in the context of the BBSome via its interaction with BBS9

**Fig 5:**
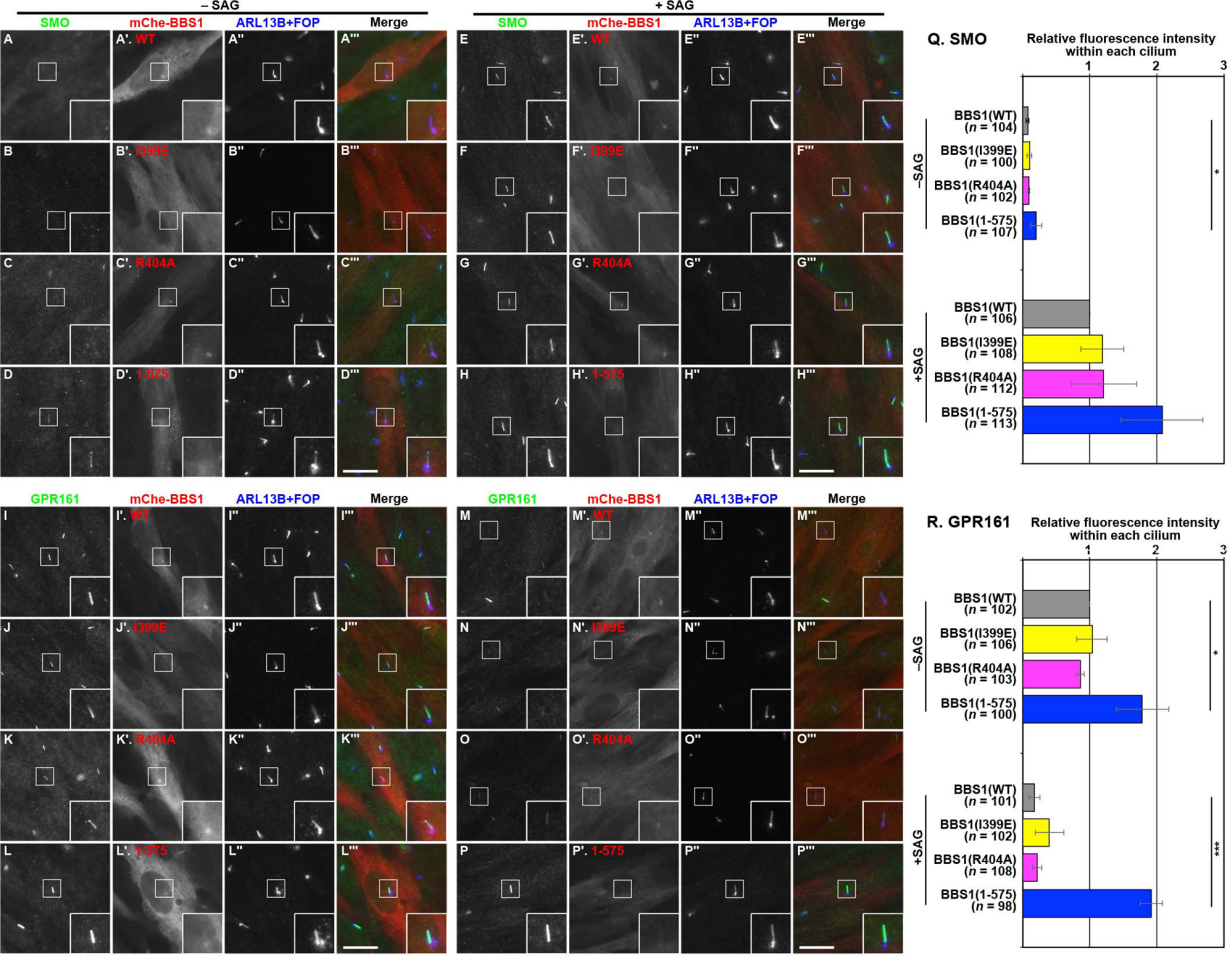
Rescue of SMO and GPR161 localization in *BBS1*-KO cells upon the expression of WT and mutant BBS1. The #B1-1-23 cell line stably expressing mChe-fused BBS1(WT) (A, E, I, and M), BBS1(I399E) (B, F, J, and N), BBS1(R404A) (C, G, K, and O), or BBS1(1–575) (D, H, L, and P) were cultured and treated with SAG as described in the legend for Fig 4, and triple immunostained for either SMO (A–H) or GPR161 (I–P), and ARL13B and FOP (A′′–P′′). (M and N) Relative staining intensities for SMO and GPR161 were estimated and expressed as described in the legend for Fig 4. Values are means ± SD of three independent experiments. In each set of experiments, 31–44 (M) and 31–45 (U) ciliated cells were analyzed, and the total numbers of ciliated cells analyzed (*n*) are shown. *, *p* < 0.05; ***, *p* < 0.0001 (one-way ANOVA followed by Tukey post-hoc analysis).

To exclude the potential off-target effects of the CRISPR/Cas9 system, we then performed rescue experiments. *BBS1*-KO cells were infected with a lentiviral vector for the stable expression of mChe-tagged BBS1(WT) or its mutant. In contrast to the *BBS1*-KO (#B1-1-23) cell line without exogenous BBS1 expression (Fig 4B, 4E, 4H and 4K), SMO was excluded from and GPR161 was localized within cilia (Fig 5A and 5I; also see Fig 5Q and 5R) in the #B1-1-23 cell line expressing mChe-BBS1(WT) under basal conditions, as observed in control cells (Fig 4A and 4G). Upon stimulation with SAG, SMO entered and GPR161 exited cilia (Fig 5E and 5M; also see Fig 5Q and 5R) in the mChe-BBS1(WT)–expressing *BBS1*-KO cell line, similarly to in control cells (Fig 4D and 4J). Thus, the impaired localization of SMO and GPR161 in the *BBS1*-KO cell line under both basal and SAG-stimulated conditions was rescued by the exogenous expression of BBS1(WT).

In striking contrast, the exogenous expression of mChe-tagged BBS1(1–575), which cannot interact with BBS9, did not restore the normal localization of SMO or GPR161 in the *BBS1*-KO cell line; a substantial, although low, level of SMO was found within cilia under basal conditions (Fig 5D; also see Fig 5Q), and an increased level of GPR161 within cilia was found under basal conditions, and the level was maintained even when the KO cells were stimulated with SAG (Fig 5P; also see Fig 5R). These data indicate that the interaction of BBS1 with BBS9, in other words, the integrity of the BBSome core subcomplex (see S1 Fig), is essential for BBSome function to mediate retrograde trafficking of ciliary GPCRs and/or their exit from cilia.

### ARL6 interacts with the BBSome via BBS1 with the aid of BBS9

In contrast to the failed recovery of GPCR retrograde trafficking by the expression of BBS1(1–575), in *BBS1*-KO cells exogenously expressing a BBS1 mutant defective in ARL6 binding substantially rescued the abnormal localization of SMO and GPR161. Namely, in the *BBS1*-KO cell line expressing mChe-BBS1(I399E) or mChe-BBS1(R404A), SMO was excluded from cilia under basal conditions (Fig 5B and 5C; also see Fig 5Q). Ciliary exit of GPR161 upon SAG treatment was promoted in *BBS1*-KO cells expressing mChe-BBS1(I399E) or mChe-BBS1(R404A) (Fig 5N and 5O), compared with those expressing mChe-BBS1(1–575) (Fig 5P; also see Fig 5R). Thus, these BBS1 point mutants appeared to be functional, at least partly, with regard to GPCR trafficking, even though they are defective in ARL6 binding (Fig 2C and 2D). Given that ARL6 demonstrates a binary interaction only with BBS1 among the BBSome subunits (S4 Fig), the results of these rescue experiments were intriguing.

**Fig 6:**
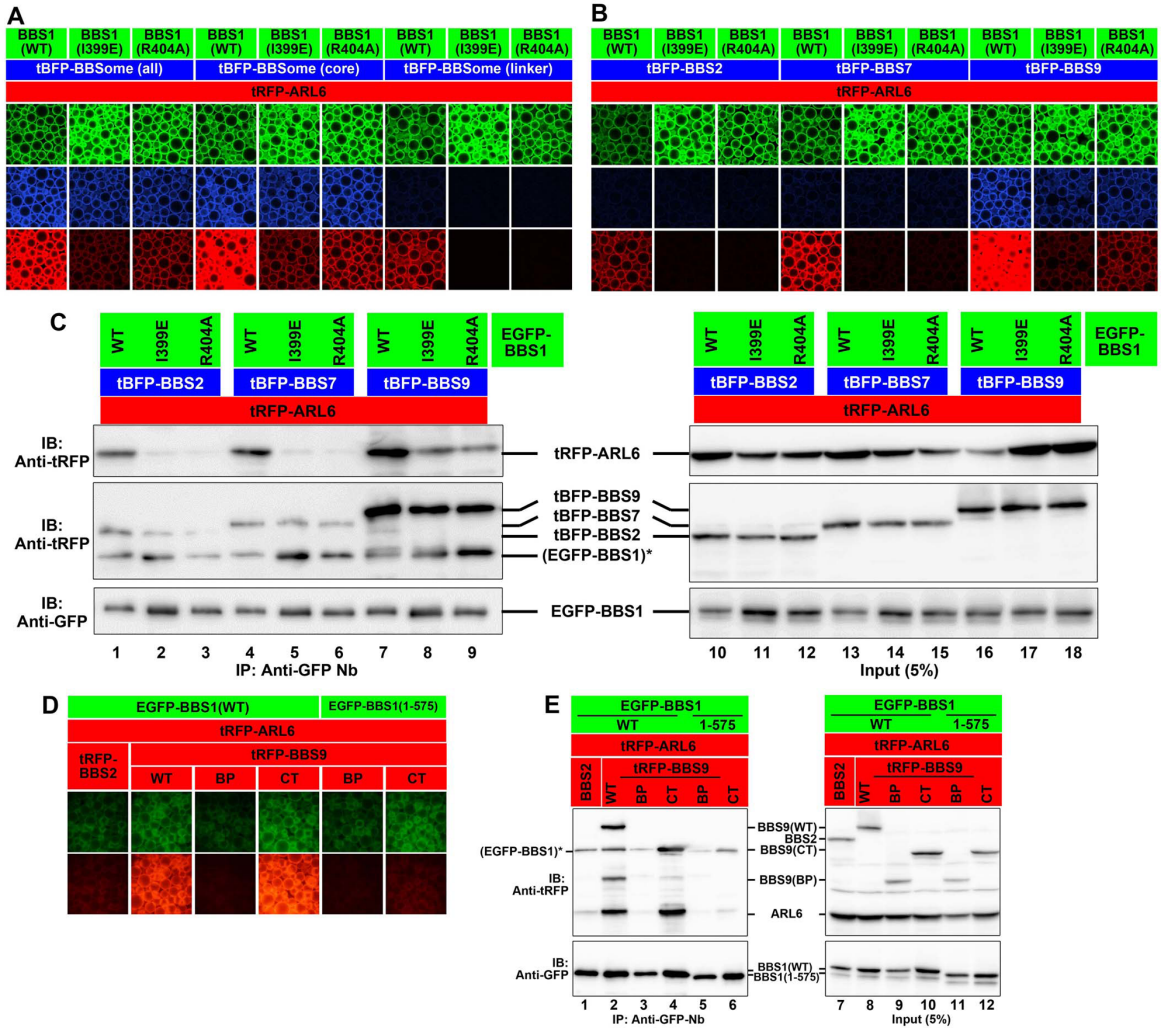
The ARL6–BBS1 interaction is strengthened by BBS9. (A) HEK293T cells were transfected with expression vectors for an EGFP-fused BBS1 construct, as indicated, and tRFP-ARL6∆N15(Q73L) together with tBFP-fusion vectors for all BBSome subunits (excepting BBS1), all core subunits (excepting BBS1), or all linker subunits. Lysates prepared from the transfected cells were processed for the VIP assay. Beads bearing fluorescent fusion proteins were observed using an A1R-MP confocal laser-scanning microscope (Nikon). (B and C) HEK293T cells were transfected with expression vectors for an EGFP-fused BBS1 construct as indicated and tRFP-ARL6∆N15(Q73L) together with tBFP-fused BBS2, BBS7, or BBS9. Lysates prepared from the transfected cells were processed for the VIP assay (B) or immunoblotting analysis (C) with anti-tRFP antibody, which reacts with tRFP and tBFP, and very weakly cross-reacts with EGFP (indicated by an asterisk) and with an anti-GFP antibody. (D and E) Lysates prepared from HEK293T cells cotransfected with expression vectors for an EGFP-fused BBS1 construct as indicated, and tRFP-ARL6∆N15(Q73L) together with tRFP-fused BBS2 or a BBS9 construct as indicated were processed for the VIP assay (D) or immunoblotting analysis (E) with anti-tRFP antibody and anti-GFP antibody. The anti-tRFP antibody very weakly cross-reacts with EGFP (indicated by an asterisk).

In an attempt to address the apparent contradiction of the data obtained by the rescue experiments of the *BBS1*-KO cells (Fig 5) with the biochemical interaction data (Fig 2C and 2D), we took advantage of the VIP assay to analyze whether the ARL6–BBS1 interaction can be enhanced in the presence of other BBSome subunit(s). For this purpose, tRFP-fused ARL6∆N15(Q73L) and either EGFP-fused BBS1(WT), BBS1(I399E), or BBS1(R404A) were co-expressed with tBFP fusions of all the other BBSome subunits, other core subunits (BBS2, BBS7, and BBS9), or linker subunits (BBS4, BBS8, and BBS18) in HEK293T cells. As shown in Fig 6A, the interaction of EGFP-BBS1(WT) with tRFP-ARL6 appeared to be promoted in the presence of tBFP-fused all the other BBSome subunits (BBS2/BBS4/BBS5/BBS7/BBS8/BBS9/BBS18; Fig 6A, column 1) or other core subunits (BBS2/BBS7/BBS9; column 4) compared with that in the presence of tBFP-fused linker subunits (BBS4/BBS8/BBS18) (column 7), the latter which does not demonstrate a direct interaction with BBS1. Importantly, substantial interaction of BBS1(I399E) or BBS1(R404A) with ARL6 was detectable in the presence of all other BBSome subunits or all other core subunits, but not in the presence of linker subunits (compare columns 1‒6 with 7‒9).

We then investigated which core subunit(s) can enhance the ARL6–BBS1 interaction. As shown in Fig 6B, the interaction between EGFP-BBS1(WT) and tRFP-ARL6 was substantially enhanced in the presence of tBFP-BBS9 (column 7), but not in the presence of tBFP-BBS2 (column 1) or tBFP-BBS7 (column 4). Furthermore, tBFP-BBS9 made the interaction between tRFP-ARL6 and EGFP-BBS1(I399E) or EGFP-BBS1(R404A) (column 8 and 9) detectable. The VIP data were confirmed by conventional immunoblotting. tBFP-BBS9 substantially increased the amount of tRFP-ARL6 coimmunoprecipitated with EGFP-BBS1(WT), as compared with tBFP-BBS2 and tBFP-BBS7 (Fig 6C, top panel; compare lane 7 with lanes 1 and 4). Furthermore, tBFP-BBS9 also led to an increase in the amount of tRFP-ARL6 coprecipitated with EGFP-BBS1(I399E) or EGFP-BBS1(R404A) (middle panel; compare lanes 8 and 9 with lanes 2 and 5, and lanes 3 and 6, respectively).

The data shown in Fig 1B indicate that the BBS9 CT region interacts with the BBS1 CT region. We then examined whether the BBS9 CT region is sufficient for promoting the ARL6 interaction with BBS1. As shown in Fig 6D and 6E, not only the WT construct (lane 2) but also the CT region (lane 4) of BBS9 considerably enhanced the interaction of BBS1(WT) with ARL6. The enhancement of the ARL6–BBS1 interaction by BBS9 was confirmed to be mediated by the interaction of BBS9 with BBS1, since the BBS9 CT region did not promote the interaction of ARL6 with BBS1(1–575) (compare lane 6 with lane 4), which lacks the BBS9-binding ability (Fig 2B).

These VIP and immunoblotting data suggest that BBS9 can reinforce the interaction of ARL6 with BBS1, although it does not directly interact with ARL6 (see Discussion). Furthermore, these data can explain why BBS1(I399E) and BBS1(R404A) can rescue, at least partially, the *BBS1*-KO phenotype (Fig 5), although neither of the BBS1 mutants forms a binary interaction with ARL6 (Fig 2C and 2D; and Fig 6B and 6C, lanes 2 and 3, and lanes 4 and 5) (see Discussion).

## Discussion

Protein trafficking within cilia is mediated by the IFT machinery composed of large protein complexes. The BBSome consists of eight BBS proteins encoded by causative genes of BBS, and has been implicated in the trafficking of ciliary membrane proteins, including GPCRs, by connecting the IFT machinery and cargo GPCRs. The membrane recruitment and coat-like assembly of the BBSome to promote cargo trafficking has been proposed to be regulated by the Arf-like small GTPase ARL6/BBS3, through its interaction with the BBS1 subunit. Using the VIP-based method, we here systematically investigated how the BBSome core subcomplex composed of BBS1, BBS2, BBS7, and BBS9 assembles and interacts with ARL6 (Fig 1). The data presented here showed that the CT regions containing the GAE and PF domains, but not the BP domains (except for that of BBS1) of these core subunits mainly participate in the assembly of the core subcomplex (Fig 1G). In other words, the BP domains of the core subunits are free from core subcomplex assembly. Taking into account the fact that the BP domains of the α-COP and δ-COP subunits of the COPI complex are responsible for recognition of cargo molecules [33,34], it is tempting to speculate that the BP domains of the BBSome subunits participate in cargo recognition, although our attempts to find interactions between the BBSome and candidate cargo molecules have so far been unsuccessful. While this manuscript was in preparation, Klink *et al*. reported that a recombinant BBSome semi-complex, which contained BBS1 and BBS9 but lacked BBS2 and BBS7, bound *in vitro* to synthetic peptides derived from ciliary GPCRs, SMO and SSTR3 [37]. Given that, in the genomes of *Drosophila* species, the BBS2 and BBS7 genes are absent [38], BBS1 and/or BBS9 might play pivotal roles in cargo recognition, although it remains possible that other subunits also play some role.

Unexpectedly, our VIP-based analysis, supported by conventional immunoblotting analysis, also demonstrated that although BBS1 directly interacts with ARL6 via its BP domain as shown by a previous crystallographic study [32], the ARL6–BBS1 interaction can be indirectly strengthened by BBS9 (Fig 6B and 6C). In view of the facts that BBS1 interacts with BBS9 via its CT region (Fig 1B) and that BBS9 did not show a direct interaction with ARL6 (S4 Fig), how BBS9 supports the ARL6-BBS1 interaction is an interesting issue to address. One possible explanation is that the BBS1 protein on its own adopts a closed conformation, but upon binding of BBS9 to its CT region, the BBS1 protein undergoes a change in conformation so that ARL6 is now accessible to its BP domain. In support of this speculation, the interaction of ARL6 with the BP domain construct of BBS1 appears to be stronger than that with the BBS1(WT) construct (Fig 1F). Another possibility is that BBS9 can somehow stabilize the ARL6–BBS1 dimer. If so, formation of the ARL6–BBS1 dimer, and subsequent BBS9 binding, can trigger the assembly of the whole BBSome complex. In any case, our data suggest that the ARL6–BBS1 interaction is maximally functional in the context of the BBSome complex.

In this study, we also established *BBS1*-KO RPE1 cells and showed that the absence of BBS1 impairs retrograde trafficking and/or export of GPR161, and possibly SMO (Fig 4). This phenotype is in line with that reported for *ARL6*-KO cells [20,21]. The impaired trafficking of these ciliary GPCRs was rescued by the exogenous expression of BBS1(WT), but not by its mutant, BBS1(1–575), defective in BBS9 binding due to the lack of only 18-amino acids from the C-terminus (Fig 5). As BBS1(1–575) retains the ability to interact with BBS7 and ARL6, the data of rescue experiments indicate that the integrity of the whole BBSome complex is crucial for its role in ciliary protein trafficking.

On the other hand, two BBS1 mutants, BBS1(I399E) and BBS1(R404A), which are defective in the binary interaction with ARL6 (Fig 2B and 2C)[32], were unexpectedly found to rescue the impaired GPCR trafficking in *BBS1*-KO cells (Fig 5). Given that *ARL6*-KO cells [20,21] show apparently the same phenotype as that of *BBS1*-KO cells (Fig 5), it was intriguing that the BBS1 mutants defective in ARL6 binding were able to restore the impaired GPCR trafficking in *BBS1*-KO cells. However, we finally found that the BBS1 mutants demonstrate a substantial, although limited, interaction with ARL6 in the presence of BBS9, as described above. Therefore, how ARL6 is implicated in BBSome function; namely, whether it regulates the assembly of the BBSome or is a stoichiometric component of the BBSome will be an interesting issue to address in the future, although these roles are not mutually exclusive.

## Supporting information

S1 Fig Schematic representation of the architecture of the BBSome predicted from our previous study. (TIF)

S2 Fig Genomic PCR and sequence analyses of the *BBS1*-KO cell lines. (A and C) Genomic DNA was extracted from control hTERT-RPE1 cells and form the *BBS1*-KO cell lines, #B1-1-23 (A) and #B1-2-21 (C), established using donor knock-in vectors containing target sequences 1 and 2, respectively. The DNA was subjected to PCR using the primer sets as indicated (see S3 Table) in an attempt to detect alleles with a small indel or no insertion (a and a′), or with forward (b and b′) or reverse (c and c′) integration of the donor knock-in vector. (B and D) Alignment of allele sequences of the B1-1-23 (B) and B1-2-21 (D) cell lines determined by direct sequencing of the genomic PCR products. Red and black lines indicate the target sequences and PAM sequence, respectively, and blue arrows indicate the direction of donor vector integration.
(TIF)

S3 Fig Ciliogenesis is not affected in the absence of BBS1. Percentages of cells with cilia (A) and the length of cilia (B) in the data shown in Fig 3A–3C, were measured and expressed as bar graphs. Values are means ± SD of three independent experiments. In each set of experiments, 34–60 (A) and 31–51 (B) cells were observed, and the total numbers of ciliated cells observed (*n*) are shown.
(TIF)

S4 Fig ARL6 can interact only with BBS1 among the BBSome subunits. Lysates prepared from HEK293T cells coexpressing EGFP-ARL6∆N15(Q73L) and each of the BBSome subunits fused to mChe were subjected to the VIP assay.
(TIF)

S1 Table Plasmid vectors used in this study. (DOCX)

S2 Table Antibodies used in this study. (DOCX)

S3 Table Oligo DNAs used in this study. (DOCX)

## References

[1] Briscoe J, Thérond PP (2013) The mechanisms of Hedgehog signalling and its roles in development and disease. Nat Rev Mol Cell Biol 14: 416–429. doi: 10.1038/nrm3598 23719536 10.1038/nrm3598

[2] Mukhopadhyay S, Rohatgi R (2014) G-protein-coupled receptors, Hedgehog signaling and primary cilia. Sem Cell Dev Biol 33: 63–72. 10.1016/j.semcdb.2014.05.002 PMC4130902 24845016

[3] Madhivanan K, Aguilar RC (2014) Ciliopathies: the trafficking connection. Traffic 15: 1031–1056. doi: 10.1111/tra.12195 25040720 10.1111/tra.12195 PMC4167927

[4] Braun DA, Hildebrandt F (2017) Ciliopathies. Cold Spring Harb Perspect Biol 9: a028191 doi: 10.1101/cshperspect.a028191 27793968 10.1101/cshperspect.a028191 PMC5334254

[5] Wei Q, Ling K, Hu J (2015) The essential roles of transition fibers in the context of cilia. Curr Opin Cell Biol 35: 98–105. doi: 10.1016/j.ceb.2015.04.015 25988548 10.1016/j.ceb.2015.04.015 PMC4529799

[6] Verhey KJ, Yang W (2016) Permeability barriers for generating a unique ciliary protein and lipid composition. Curr Opin Cell Biol 41: 109–116. doi: 10.1016/j.ceb.2016.05.004 27232950 10.1016/j.ceb.2016.05.004 PMC4983468

[7] Rosenbaum JL, Witman GB (2002) Intraflagellar transport. Nat Rev Mol Cell Biol 3: 813–825. doi: 10.1038/nrm952 12415299 10.1038/nrm952

[8] Sung C-H, Leroux MR (2013) The roles of evolutionary conserved functional modules in cilia-related trafficking. Nat Cell Biol 15: 1387–1397. doi: 10.1038/ncb2888 24296415 10.1038/ncb2888 PMC4016715

[9] Ishikawa H, Marshall WF (2011) Ciliogenesis: building the cell's antenna. Nat Rev Mol Cell Biol 12: 222–234. doi: 10.1038/nrm3085 21427764 10.1038/nrm3085

[10] Taschner M, Lorentzen E (2016) The intraflagellar transport machinery. Cold Spring Harb Perspect Biol 8: a028092 doi: 10.1101/cshperspect.a028092 27352625 10.1101/cshperspect.a028092 PMC5046692

[11] Nakayama K, Katoh Y (2018) Ciliary protein trafficking mediated by IFT and BBSome complexes with the aid of kinesin-2 and dynein-2 motors. J Biochem 163: 155–164. doi: 10.1093/jb/mvx087 29272450 10.1093/jb/mvx087

[12] Williams CL, McIntyre JC, Norris SR, Jenkins PM, Zhang L, Pei Q, et al (2014) Direct evidence for BBSome-associated intraflagellar transport reveals distinct properties of native mammalian cilia. Nat Commun 5: 5813 doi: 10.1038/ncomms6813 25504142 10.1038/ncomms6813 PMC4284812

[13] Taschner M, Weber K, Mourão A, Vetter M, Awasthi M, Stiegler M, et al (2016) Intraflagellar transport proteins 172, 80, 57, 54, 38, and 20 form a stable tubulin-binding IFT-B2 complex. EMBO J 35: 773–790. doi: 10.15252/embj.201593164 26912722 10.15252/embj.201593164 PMC4818760

[14] Katoh Y, Terada M, Nishijima Y, Takei R, Nozaki S, Hamada H, et al (2016) Overall architecture of the intraflagellar transport (IFT)-B complex containing Cluap1/IFT38 as an essential component of the IFT-B peripheral subcomplex. J Biol Chem 291: 10962–10975. doi: 10.1074/jbc.M116.713883 26980730 10.1074/jbc.M116.713883 PMC4900248

[15] Boldt K, van Reeuwijk J, Lu Q, Koutroumpas K, Nguyen TM, Texier Y, et al (2016) An organelle-specific protein landscape identifies novel diseases and molecular mechanisms. Nat Commun 7: 11491 doi: 10.1038/ncomms11491 27173435 10.1038/ncomms11491 PMC4869170

[16] Hirano T, Katoh Y, Nakayama K (2017) Intraflagellar transport-A complex mediates ciliary entry and retrograde trafficking of ciliary G protein-coupled receptors. Mol Biol Cell 28: 429–439. doi: 10.1091/mbc.E16-11-0813 27932497 10.1091/mbc.E16-11-0813 PMC5341726

[17] Katoh Y, Nozaki S, Hartanto D, Miyano R, Nakayama K (2015) Architectures of multisubunit complexes revealed by a visible immunoprecipitation assay using fluorescent fusion proteins. J Cell Sci 128: 2351–2362. doi: 10.1242/jcs.168740 25964651 10.1242/jcs.168740

[18] Nachury MV, Loktev AV, Zhang Q, Westlake CJ, Peränen J, Merdes A, et al (2007) A core complex of BBS proteins cooperates with the GTPase Rab8 to promote ciliary membrane biogenesis. Cell 129: 1201–1213. doi: 10.1016/j.cell.2007.03.053 17574030 10.1016/j.cell.2007.03.053

[19] Jin H, White SR, Shida T, Schulz S, Aguilar M, Gygi SP, et al (2010) The conserved Bardet-Biedl syndrome proteins assemble a coat that traffics membrane proteins to cilia. Cell 141: 1208–1219. doi: 10.1016/j.cell.2010.05.015 20603001 10.1016/j.cell.2010.05.015 PMC2898735

[20] Zhang Q, Nishimura D, Seo S, Vogel T, Morgan DA, Searby C, et al (2011) Bardet-Biedl syndrome 3 (Bbs3) knockout mouse model reveals common BBS-associated phenotypes and Bbs3 unique phenotypes. Proc Natl Acad Sci USA 108: 20678–20683. doi: 10.1073/pnas.1113220108 22139371 10.1073/pnas.1113220108 PMC3251145

[21] Liew GM, Ye F, Nager AR, Murphy JP, Lee JSH, Aguiar M, et al (2014) The intraflagellar transport protein IFT27 promotes BBSome exit from cilia through the GTPase ARL6/BBS3. Dev Cell 31: 265–278. doi: 10.1016/j.devcel.2014.09.004 25443296 10.1016/j.devcel.2014.09.004 PMC4255629

[22] Seo S, Zhang Q, Bugge K, Breslow DK, Searby CC, Nachury MV, et al (2011) A novel protein LZTFL1 regulates ciliary trafficking of the BBSome and Smoothened. PLoS Genet 7: e1002358 doi: 10.1371/journal.pgen.1002358 22072986 10.1371/journal.pgen.1002358 PMC3207910

[23] Eguether T, San Agustin JT, Keady BT, Jonassen JA, Liang Y, Francis R, et al (2014) IFT27 links the BBSome to IFT for maintenance of the ciliary signaling compartment. Dev Cell 21: 279–290. 10.1016/j.devcel.2014.09.011 PMC4254547 25446516

[24] Nishijima Y, Hagiya Y, Kubo T, Takei R, Katoh Y, Nakayama K (2017) RABL2 interacts with the intraflagellar transport B complex and CEP19 and participates in ciliary assembly. Mol Biol Cell 28: 1652–1666. doi: 10.1091/mbc.E17-01-0017 28428259 10.1091/mbc.E17-01-0017 PMC5469608

[25] Katoh Y, Michisaka S, Nozaki S, Funabashi T, Hirano T, Takei R, et al (2017) Practical method for targeted disruption of cilia-related genes by using CRISPR/Cas9-mediated homology-independent knock-in system. Mol Biol Cell 28: 898–906. doi: 10.1091/mbc.E17-01-0051 28179459 10.1091/mbc.E17-01-0051 PMC5385939

[26] Funabashi T, Katoh Y, Michisaka S, Terada M, Sugawa M, Nakayama K (2017) Ciliary entry of KIF17 is dependent on its binding to the IFT-B complex via IFT46-IFT56 as well as on its nuclear localization signal. Mol Biol Cell 28: 624–633. doi: 10.1091/mbc.E16-09-0648 28077622 10.1091/mbc.E16-09-0648 PMC5328621

[27] Nozaki S, Katoh Y, Terada M, Michisaka S, Funabashi T, Takahashi S, et al (2017) Regulation of ciliary retrograde protein trafficking by the Joubert syndrome proteins ARL13B and INPP5E. J Cell Sci 130: 563–576. doi: 10.1242/jcs.197004 27927754 10.1242/jcs.197004

[28] Hsu PD, Scott DA, Weinstein JA, Ran FA, Konermann S, Agarwala V, et al (2013) DNA targeting specificity of RNA-guided Cas9 nucleases. Nat Biotechnol 31: 827–832. doi: 10.1038/nbt.2647 23873081 10.1038/nbt.2647 PMC3969858

[29] Takahashi S, Kubo K, Waguri S, Yabashi A, Shin H-W, Katoh Y, et al (2012) Rab11 regulates exocytosis of recycling vesicles at the plasma membrane. J Cell Sci 125: 4049–4057. doi: 10.1242/jcs.102913 22685325 10.1242/jcs.102913

[30] Thomas S, Ritter B, Verbich D, Sanson C, Bourbonnière L, McKinney R.A, et al (2009) Intersectin regulates dendritic spine development and somatodendritic endocytosis but not synaptic vesicle recycling in hippocampal neurons. J Biol Chem 284: 12410–12419. doi: 10.1074/jbc.M809746200 19258322 10.1074/jbc.M809746200 PMC2673308

[31] Knockenhauer KE, Schwartz TU (2015) Structural characterization of Bardet-Biedl syndorme 9 protein (BBS9). J Biol Chem 290: 19569–19583. doi: 10.1074/jbc.M115.649202 26085087 10.1074/jbc.M115.649202 PMC4528124

[32] Mourão A, Nager AR, Nachury MV, Lorentzen E (2014) Structural basis for membrane targeting of the BBSome by ARL6. Nat Struct Mol Biol 21: 1035–1041. doi: 10.1038/nsmb.2920 25402481 10.1038/nsmb.2920 PMC4255524

[33] Ma W, Goldberg J (2013) Rules for the recognition of dilysine retrieval motifs by coatomer. EMBO J 32: 926–937. doi: 10.1038/emboj.2013.41 23481256 10.1038/emboj.2013.41 PMC3616288

[34] Jackson LP, Lewis M, Kent HM, Edeling MA, Evans PR, Duden R, et al (2012) Molecular basis for recognition of dilysine trafficking motifs by COPI. Dev Cell 23: 1255–1262. doi: 10.1016/j.devcel.2012.10.017 23177648 10.1016/j.devcel.2012.10.017 PMC3521961

[35] Nakayama K, Wakatsuki S (2003) The structure and function of GGAs, the traffic controllers at the TGN sorting crossroads. Cell Struct Funct 28: 431–442. 14745135 10.1247/csf.28.431

[36] Woodsmith J, Apelt L, Casado-Medrano V, Özkan Z, Timmermann B, Stelzl U (2017) Protein interaction perturbation profiling at amino-acid resolution. Nat Methods 14: 1213–1221. doi: 10.1038/nmeth.4464 29039417 10.1038/nmeth.4464

[37] Klink BU, Zent E, Juneja P, Kuhlee A, Rausner S, Wittinghofer A (2017) A rocombinant BBSome core complex and how it interacts with ciliary cargo. eLife 6: e27434 doi: 10.7554/eLife.27434 29168691 10.7554/eLife.27434 PMC5700813

[38] Shida T, Cueva JG, Xu Z, Goodman MB, Nachury MV (2010) The major α-tubulin K40 acetyltransferase αTAT1 promotes rapid ciliogenesis and efficient mechanosensation. Proc Natl Acad Sci USA 107: 21517–21522. doi: 10.1073/pnas.1013728107 21068373 10.1073/pnas.1013728107 PMC3003046

