# Supplementary figures and images for: BBS1 is involved in retrograde trafficking of ciliary GPCRs in the context of the BBSome complex
Source: PLoS One. 2018 Mar 28;13(3):e0195005. doi: 10.1371/journal.pone.0195005 (PMC5874067; doi:10.1371/journal.pone.0195005)

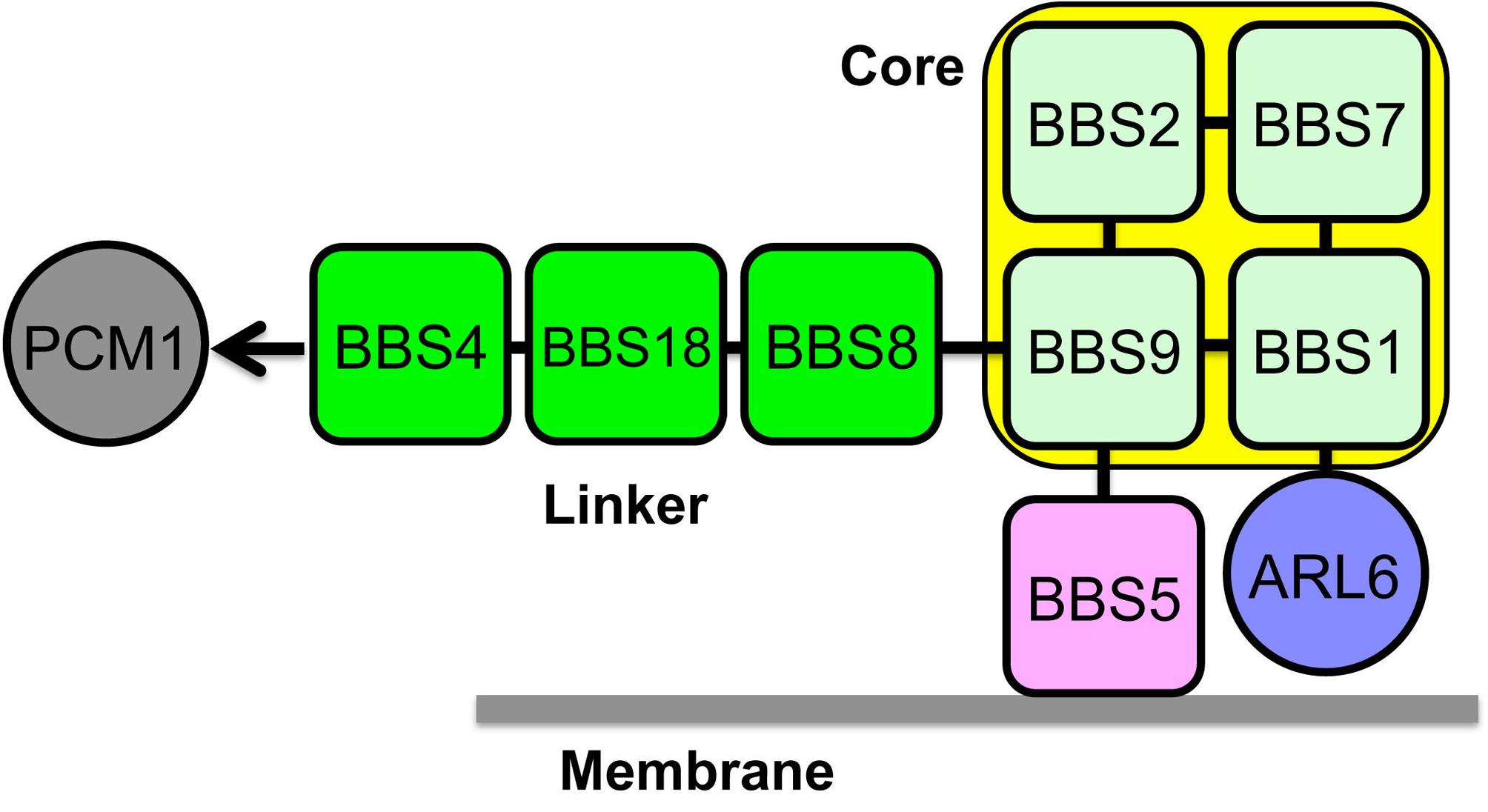

Supplement: S1 Fig — (TIF) [file pone.0195005.s001.tif]

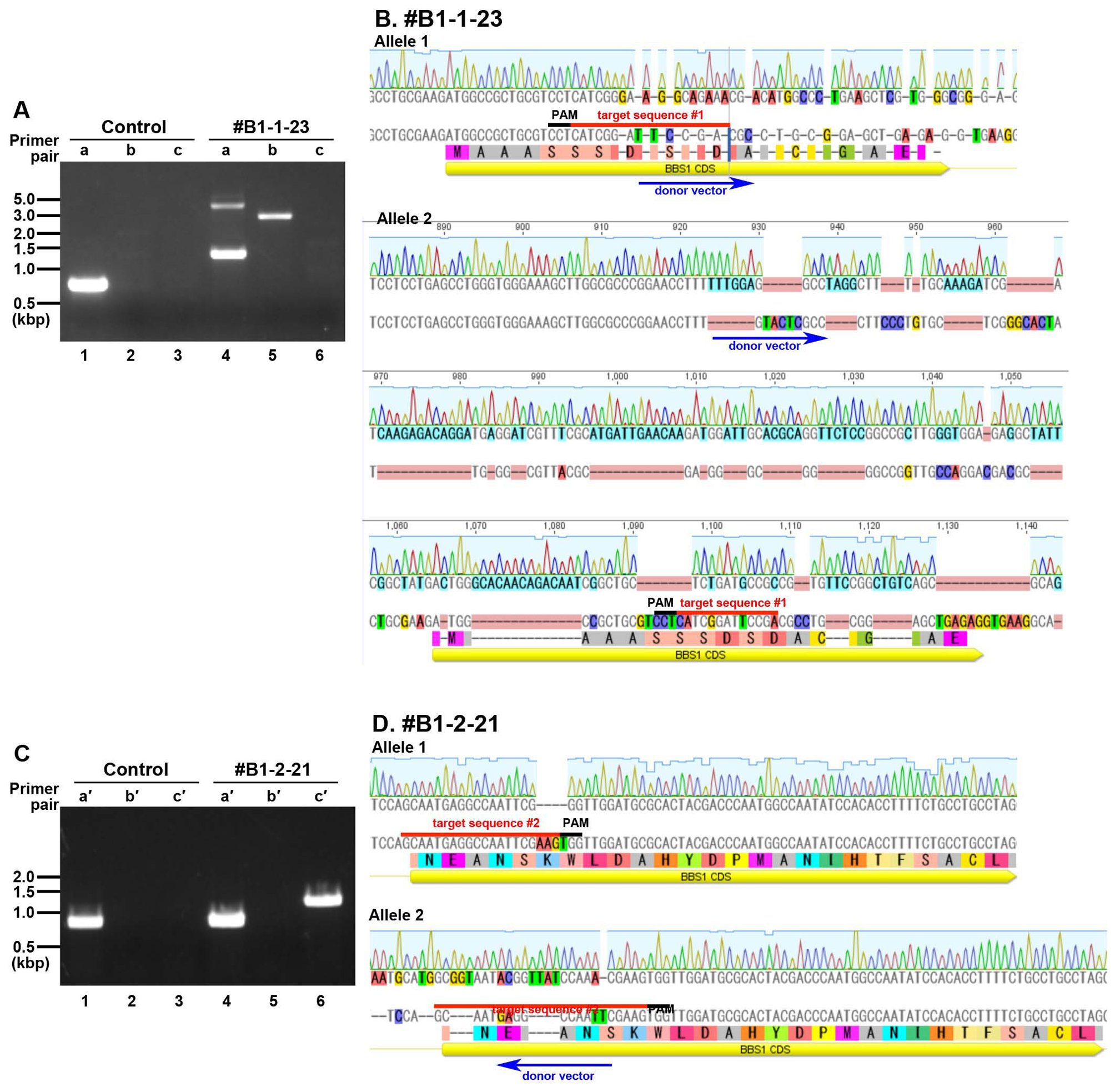

Supplement: S2 Fig — (A and C) Genomic DNA was extracted from control hTERT-RPE1 cells and form the BBS1-KO cell lines, #B1-1-23 (A) and #B1-2-21 (C), established using donor knock-in vectors containing target sequences 1 and 2, respectively. The DNA was subjected to PCR using the primer sets as indicated (see S3 Table) in an attempt to detect alleles with a small indel or no insertion (a and a′), or with forward (b and b′) or reverse (c and c′) integration of the donor knock-in vector. (B and D) Alignment of allele sequences of the B1-1-23 (B) and B1-2-21 (D) cell lines determined by direct sequencing of the genomic PCR products. Red and black lines indicate the target sequences and PAM sequence, respectively, and blue arrows indicate the direction of donor vector integration. (TIF) [file pone.0195005.s002.tif]

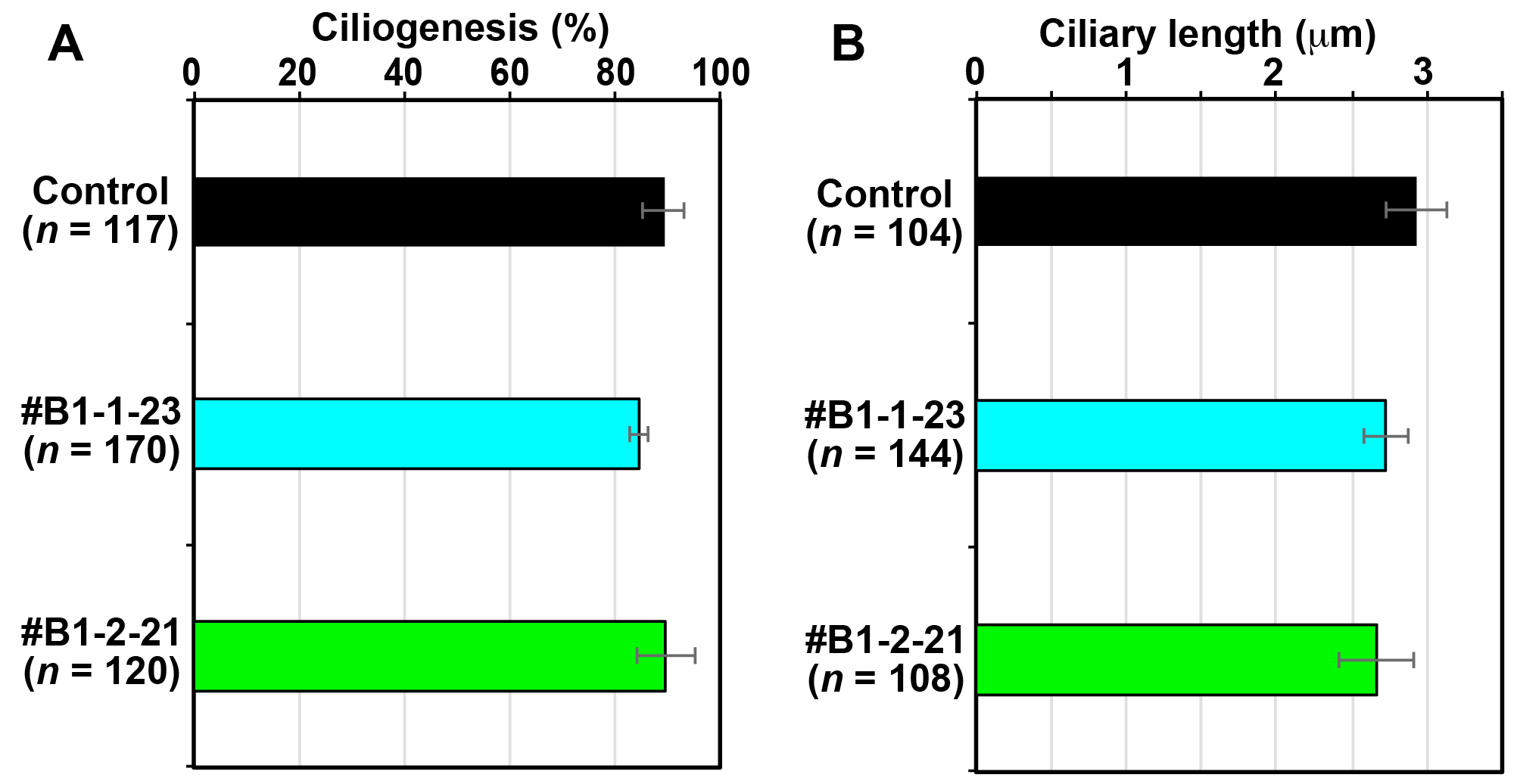

Supplement: S3 Fig — Percentages of cells with cilia (A) and the length of cilia (B) in the data shown in Fig 3A–3C, were measured and expressed as bar graphs. Values are means ± SD of three independent experiments. In each set of experiments, 34–60 (A) and 31–51 (B) cells were observed, and the total numbers of ciliated cells observed (n) are shown. (TIF) [file pone.0195005.s003.tif]

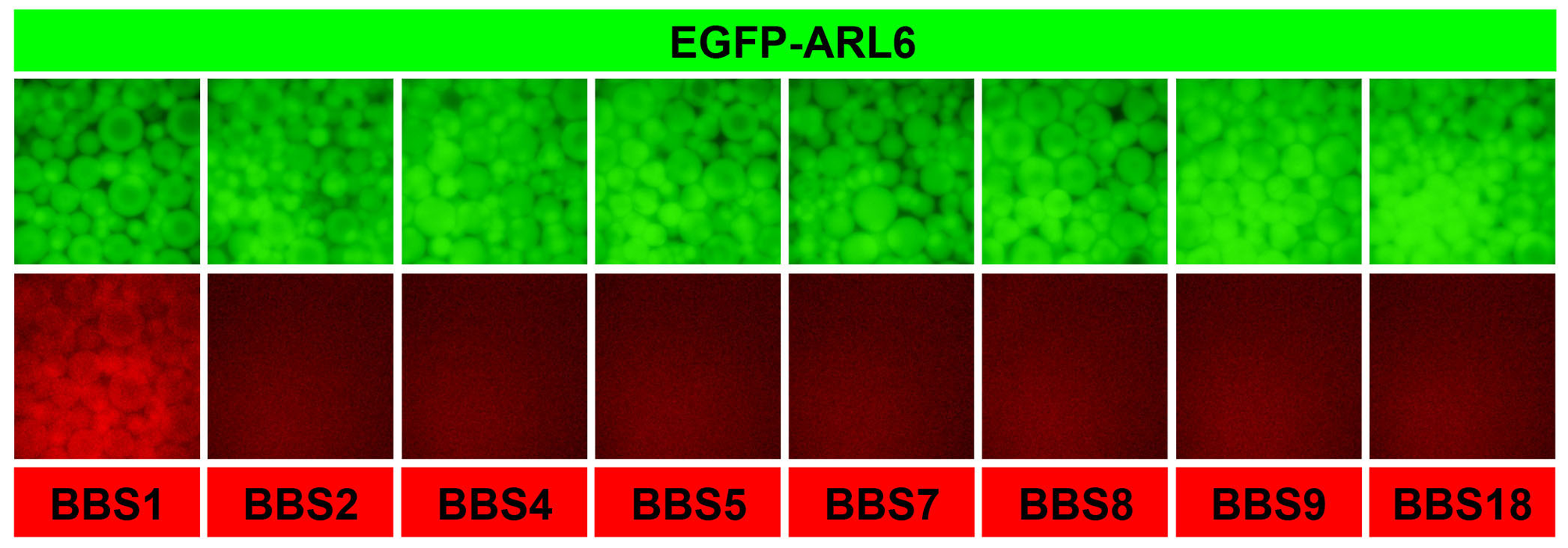

Supplement: S4 Fig — Lysates prepared from HEK293T cells coexpressing EGFP-ARL6∆N15(Q73L) and each of the BBSome subunits fused to mChe were subjected to the VIP assay. (TIF) [file pone.0195005.s004.tif]
