# Supplementary material for: BBS1 is involved in retrograde trafficking of ciliary GPCRs in the context of the BBSome complex
Source: PLoS One. 2018 Mar 28;13(3):e0195005. doi: 10.1371/journal.pone.0195005 (PMC5874067; doi:10.1371/journal.pone.0195005)
Supplement: S1 Table — (DOCX) [file pone.0195005.s005.docx]

**S1 Table. Plasmid vectors used in this study**

| No | Vector | Insert | Reference |
| --- | --- | --- | --- |
| 1 | pcDNA3-EGFP-C | Human BBS1 | ([Katoh *et al.*, 2015](#_ENREF_1)) |
| 2 | pcDNA3-EGFP-C | Human BBS1(BP, 1-430) | This study |
| 3 | pcDNA3-EGFP-C | Human BBS1(CT, 431-593) | This study |
| 4 | pEGFP-C1 | Human BBS1(I399E) | This study |
| 5 | pEGFP-C1 | Human BBS1(R404A) | This study |
| 6 | pEGFP-C1 | Human BBS1(da, 1-430, 483-593) | This study |
| 7 | pEGFP-C1 | Human BBS1(1-575) | This study |
| 8 | pTagRFP-T-C | Human BBS1 | ([Katoh *et al.*, 2015](#_ENREF_1)) |
| 9 | pTagRFP-T-C | Human BBS1(BP, 1-430) | This study |
| 10 | pTagRFP-T-C | Human BBS1(CT, 431-593) | This study |
| 11 | pTagRFP-T-C | Human BBS1(I399E) | This study |
| 12 | pTagRFP-T-C | Human BBS1(R404A) | This study |
| 13 | pTagRFP-T-C | Human BBS1(da, 1-430, 483-593) | This study |
| 14 | pTagRFP-T-C | Human BBS1(1-575) | This study |
| 15 | pRRLsinPPT-mCherry-C | Human BBS1 | This study |
| 16 | pRRLsinPPT-mCherry-C | Human BBS1(I399E) | This study |
| 17 | pRRLsinPPT-mCherry-C | Human BBS1(R404A) | This study |
| 18 | pRRLsinPPT-mCherry-C | Human BBS1(1-575) | This study |
| 19 | pTagBFP2-C | Human BBS1 | This study |
| 20 | pEGFP-C1 | Human BBS2 | ([Katoh *et al.*, 2015](#_ENREF_1)) |
| 21 | pEGFP-C1 | Human BBS2(BP, 1-330) | This study |
| 22 | pEGFP-C1 | Human BBS2(CT, 331-721) | This study |
| 23 | pTagRFP-T-C | Human BBS2 | ([Katoh *et al.*, 2015](#_ENREF_1)) |
| 24 | pTagRFP-T-C | Human BBS2(BP, 1-330) | This study |
| 25 | pTagRFP-T-C | Human BBS2(CT, 331-721) | This study |
| 26 | pTagBFP2-C | Human BBS2 | This study |
| 27 | pTagBFP2-C | Human BBS4 | This study |
| 28 | pTagRFP-T-C | Human BBS5 | ([Katoh *et al.*, 2015](#_ENREF_1)) |
| 29 | pTagBFP2-C | Human BBS5 | This study |
| 30 | pEGFP-C1 | Human BBS7 | ([Katoh *et al.*, 2015](#_ENREF_1)) |
| 31 | pmCherry-C1 | Human BBS7 | This study |
| 32 | pmCherry-C1 | Human BBS7(BP, 1-322) | This study |
| 33 | pmCherry-C1 | Human BBS7(CT, 323-715) | This study |
| 34 | pTagBFP2-C | Human BBS7 | ([Katoh *et al.*, 2015](#_ENREF_1)) |
| 35 | pTagRFP-T-C | Human BBS8 | ([Katoh *et al.*, 2015](#_ENREF_1)) |
| 36 | pTagBFP2-C | Human BBS8 | This study |
| 37 | pEGFP-C1 | Human BBS9 | ([Katoh *et al.*, 2015](#_ENREF_1)) |
| 38 | pEGFP-C1 | Human BBS9(BP, 1-378) | This study |
| 39 | pEGFP-C1 | Human BBS9(CT, 379-887) | This study |
| 40 | pTagRFP-T-C | Human BBS9 | ([Katoh *et al.*, 2015](#_ENREF_1)) |
| 41 | pTagRFP-T-C | Human BBS9(BP, 1-378) | This study |
| 42 | pTagRFP-T-C | Human BBS9(CT, 379-887) | This study |
| 43 | pTagBFP2-C | Human BBS9 | This study |
| 44 | pTagBFP2-C | Human BBS18 | ([Katoh *et al.*, 2015](#_ENREF_1)) |
| 45 | pEGFP-C1 | Human ARL6∆N15(Q73L) | This study |
| 46 | pTagRFP-T-C | Human ARL6∆N15(Q73L) | This study |
