# Supplementary material for: BBS1 is involved in retrograde trafficking of ciliary GPCRs in the context of the BBSome complex
Source: PLoS One. 2018 Mar 28;13(3):e0195005. doi: 10.1371/journal.pone.0195005 (PMC5874067; doi:10.1371/journal.pone.0195005)
Supplement: S2 Table — (DOCX) [file pone.0195005.s006.docx]

**S2 Table. Antibodies used in this study**

| Antibody | Manufacturer | Clone or  catalog number | Dilution (purpose) |
| --- | --- | --- | --- |
| Monoclonal mouse anti-Ac-α-tubulin | Sigma-Aldrich | 6-11B-1 | 1:1,000 (immunofluorescence) |
| Monoclonal mouse anti-γ-tubulin | Sigma-Aldrich | GTU88 | 1:1,000 (immunofluorescence) |
| Polyclonal rabbit anti-ARL13B | Proteintech | 17711-1-AP | 1:1000 (immunofluorescence) |
| Polyclonal rabbit anti-ARL6 | Proteintech | 12676-1-AP | 1:500 (immunofluorescence) |
| Polyclonal rabbit anti-BBS5 | Proteintech | 14569-1-AP | 1:500 (immunofluorescence) |
| Polyclonal rabbit anti-BBS9 | Atlas antibodies | HPA021289 | 1:1000 (immunofluorescence) |
| Polyclonal rabbit anti-IFT88 | Proteintech | 13967-1-AP | 1:200(immunofluorescence) |
| Polyclonal rabbit anti-IFT140 | Proteintech | 17460-1-AP | 1:100 (immunofluorescence) |
| Polyclonal rabbit anti-GPR161 | Proteintech | 13398-1-AP | 1:200 (immunofluorescence) |
| Polyclonal rabbit anti-SMO | Abcam | ab38686 | 1:500 (immunofluorescence) |
| Monoclonal mouse anti-ARL13B | Abcam | N295B/66 | 1:500 (immunofluorescence) |
| Monoclonal mouse anti-FOP | Abnova | 2B1 | 1:10,000 (immunofluorescence) |
| Monoclonal mouse anti-GFP | BD Biosciences | JL-8 | 1:1,000 (immunoblotting) |
| Polyclonal rabbit anti-RFP | MBL Life Science | PM005 | 1:1,000 (immunoblotting) |
| Polyclonal rabbit anti-TagRFP | Evrogen | AB233 | 1:1,000 (immunoblotting) |
| AlexaFluor-conjugated secondary | Molecular Probes | A11034, A21147, A21240 | 1:1,000 (immunofluorescence) |
| DyLight 649-conjugated secondary | Jackson ImmunoResearch | 115-495-209 | 1:3,000 (immunofluorescence) |
| Peroxidase-conjugated secondary | Jackson ImmunoResearch | 115-035-166, 111-035-144 | 1:3,000 (immunoblotting) |
