# Supplementary material for: BBS1 is involved in retrograde trafficking of ciliary GPCRs in the context of the BBSome complex
Source: PLoS One. 2018 Mar 28;13(3):e0195005. doi: 10.1371/journal.pone.0195005 (PMC5874067; doi:10.1371/journal.pone.0195005)
Supplement: S3 Table — (DOCX) [file pone.0195005.s007.docx]

**S3 Table. Oligo DNAs used in this study**

| No. | Name | Sequence |
| --- | --- | --- |
| 1 | pTagBFP-N-RV2 | 5'-CGTAGAGGAAGCTAGTAGCCAGG-3' |
| 2 | BBS1-genome#1-FW | 5'-GACAGGAGGAGTTCTTTTTGCTGC-3' |
| 3 | BBS1-genome#1-RV | 5'-AACCACTTCGAATTGGCCTCATTG-3' |
| 4 | BBS1-gRNA#1-S | 5'-CACCGCAGGCGTCGGAATCCGATG-3' |
| 5 | BBS1-gRNA#1-AS | 5'-AAACCATCGGATTCCGACGCCTGC-3' |
| 6 | BBS1-genome#2-FW | 5'-CTCGGGCACTATTGGGCGTTAC-3' |
| 7 | BBS1-genome#2-RV | 5'-AGGCTTTGTCTTAGTAGAATCCCCA-3' |
| 8 | BBS1-gRNA#2-S | 5'-CACCGCAATGAGGCCAATTCGAAG-3' |
| 9 | BBS1-gRNA#2-AS | 5'-AAACCTTCGAATTGGCCTCATTGC-3' |
